# New perspectives on epigenetic modifications and PARP inhibitor resistance in HR-deficient cancers

**DOI:** 10.20517/cdr.2022.73

**Published:** 2023-01-04

**Authors:** Rachel Bayley, Ellie Sweatman, Martin R. Higgs

**Affiliations:** Institute of Cancer and Genomic Sciences, University of Birmingham, Birmingham B15 2TT, UK.; ^#^Both authors contributed equally.

**Keywords:** Double strand break repair, histone methylation, PARP inhibitor, resistance, SETD1A, BOD1L, H3K4

## Abstract

The clinical treatment of DNA-repair defective tumours has been revolutionised by the use of poly(ADP) ribose polymerase (PARP) inhibitors. However, the efficacy of these compounds is hampered by resistance, which is attributed to numerous mechanisms including rewiring of the DNA damage response to favour pathways that repair PARP inhibitor-mediated damage. Here, we comment on recent findings by our group identifying the lysine methyltransferase SETD1A as a novel factor that conveys PARPi resistance. We discuss the implications, with a particular focus on epigenetic modifications and H3K4 methylation. We also deliberate on the mechanisms responsible, the consequences for the refinement of PARP inhibitor use in the clinic, and future possibilities to circumvent drug resistance in DNA-repair deficient cancers.

## CANCER THERAPY AND DOUBLE STRAND BREAK REPAIR

Many cancer patients will receive radiotherapy as part of their treatment^[1]^ which relies on ionising radiation (IR) to induce highly toxic lesions in the form of chromosomal DNA double-strand breaks (DSBs). DSBs represent the most lethal type of DNA damage induced by genotoxic therapy, but their programmed repair have important physiological roles in normal metabolism and immune system development. Repair of DSBs is also essential to maintain genome stability and therefore represents a vital anti-tumour barrier^[2]^.

There are two main pathways used by cells to ensure the efficient repair of DSBs, non-homologous end joining (NHEJ) and homologous recombination (HR). The choice between these two repair pathways is tightly regulated by numerous mechanisms including the cell cycle, post-translational modifications of DNA repair proteins, and interactions between DNA repair proteins and chromatin^[3]^. NHEJ is the principal DSB repair pathway and is responsible for repairing 85% of all DSBs induced by IR^[4]^. Although NHEJ is active in all phases of the cell cycle, it predominates in G1, and involves direct ligation of the two broken DNA ends. In contrast, HR requires a homologous sister chromatid as a repair template and is therefore restricted to the S and G2 cell cycle phases. HR also requires DNA end resection, which is carried out by a number of cellular nucleases including MRE11, CtIP, EXO1 and DNA2^[5,6]^. The actions of these proteins results in formation of a 3’ ssDNA tail, which is then coated with the single-stranded DNA binding protein RPA (Replication Protein A) which acts as a substrate for RAD51-mediated homology search and strand invasion. In addition to these two classical DSB repair pathways, alternative mutagenic DSB repair mechanisms have been identified. These include microhomology-mediated end joining (MMEJ; also known as alternative end-joining) and single strand annealing (SSA)^[7]^. As with HR, these alternative pathways rely on extensive end resection. However, lack of a repair template results in significant loss of genetic information in these pathways, therefore MMEJ and SSA are considered error-prone and highly mutagenic.

## BRCA1 AND 53BP1: BALANCING DSB REPAIR

The choice between HR and NHEJ is controlled by multiple factors, of which 53BP1 and BRCA1 are two of the most important. The antagonistic relationship between 53BP1 and BRCA1 controls DNA-end resection and thus dictates repair pathway choice. 53BP1 is one of the first proteins recruited to DSBs, which is mediated by the interaction between 53BP1 and two histone modifications: H4K20me2 and H2AK15Ub. Localisation of 53BP1 at DSBs protects DNA from resection via a series of downstream effectors including PTIP^[8]^, RIF1^[9-12]^, REV7^[13,14]^, and the Shieldin -CST- polα complex^[15-18]^. This pathway inhibits the localisation and activity of BRCA1 and the endonuclease CtIP to DSBs, promoting NHEJ, and maintains DSBs via fill-in of ssDNA.

In contrast, the tumour suppressor BRCA1 promotes end-resection in S and G2 and counteracts 53BP1. In part, this is mediated by post-replicative dilution of H4K20me2 on “parental” histones, promoting the recruitment of BRCA1 and its partner BARD1 to DSBs where it displaces 53BP1 from DNA ends^[19,20]^. BRCA1 also facilitates end-resection and therefore HR by promoting the actions of phosphorylated CtIP and MRE11^[21]^. In addition, BRCA1 has further roles in HR, promoting recruitment of the RAD51 recombinase to ssDNA. BRCA1 also interacts via PALB2 with BRCA2, with this tripartite complex assisting RAD51 loading and recombination^[22]^. Finally, BRCA1 and BARD1 enhance the recombinase activity of RAD51, promoting successful HR repair.

## HISTONE METHYLATION AND DNA REPAIR

Histone lysine methylation is a critical post-translational modification essential for numerous cellular processes. This modification is carried out on lysine residues within histone tails by a family of enzymes known as lysine methyltransferases (KMTs). In terms of DNA repair, several lysine methylation events are known to be required for the proper repair of DSBs^[23]^. For example, the localisation of 53BP1 to damaged chromatin requires binding of its tandem Tudor domains to di-methylated lysine 20 of histone 4 (H4K20me2)^[24-26]^. Furthermore, the binding of BARD1 to di-methylated H3K9 (H3K9me2) is required to retain BRCA1 at DSB sites to promote repair by HR^[27]^. These examples illustrate the importance of histone methylation events in the regulation and recruitment of proteins critical for DSB repair.

Methylation of lysine 4 of histone H3 (H3K4me) is most well-known as a marker for genomic regions undergoing active transcription. In yeast, H3K4me is carried out by a single methyltransferase, Set1, but in higher eukaryotes this modification is principally catalysed by the KMT2 family of enzymes^[28]^. Studies have shown that transcription is required for DSB repair and suggested that without active transcription DNA damage response (DDR) proteins are unable to efficiently localise to repair foci^[29-31]^. However, transcription can also be largely supressed at sites of DSBs despite the presence of H3K4me^[32]^, suggesting that this histone modification could also have an important transcription-independent role in DNA repair.

Several studies have examined levels of H3K4me at DSBs, yielding conflicting results. Globally, there appears to be no change in the levels of H3K4me3 following DNA damage when examined by immunoblotting^[33,34]^. However, more sensitive methods have demonstrated differences in the prevalence of this modification at DSBs. Several studies suggest that H3K4 di-and tri-methylation levels decrease following UV laser micro irradiation or in GFP-based DSB repair reporter assays. This is attributed to increased activity of various lysine demethylases (KDMs) that act on H3K4, including KDM1A, KDM5A and KDM5B^[35-37]^. In contrast, other studies demonstrate an increase in H3K4me3 at DSBs, the removal of which by KDM5B is required to allow recruitment of DNA repair factors^[38]^. We recently used chromatin immunoprecipitation (ChIP) to measure levels of histone methylation surrounding newly-formed DSBs induced on a Lac-operator by mCherry-lacI-FokI. These studies revealed an increase in H3K4me3 following DSB induction^[39]^. Collectively these data all indicate an important role for H3K4me in DSB repair, however their conflicting findings suggest that results could be dependent upon type of DNA damage induced and the methods used to detect this modification. Interestingly, H3K4 methylation is also important for other types of DNA repair, as loss of H3K4me at replication forks during replication stress induces genome instability by allowing degradation of DNA^[40]^.

## H3K4 METHYLATION AND HR/NHEJ

Analysis of DSBs undergoing repair by NHEJ or HR (classified by the proteins bound to these breaks) first identified that HR-competent chromatin is enriched in H3K4me2^[41]^. In support of this, favouring HR-mediated repair by treating cells with an inhibitor of DNA-PKcs increases levels of H3K4me at DSBs induced by the yeast rare-cutting endonuclease ISceI^[42]^. These studies on regions of “open” chromatin initially suggested that H3K4me may promote HR-mediated repair.

Recently, we have significantly revised thFeither BRCA1 or BRCA2 are associated with susceptibility to multipleese findings by identifying an important role for H3K4 methylation in facilitating RIF1-dependent NHEJ^[39]^. We showed that loss of SETD1A, a member of the KMT2 family of methyltransferases, or its cofactor BOD1L, significantly impairs RIF1 localisation to DSBs and their subsequent repair by NHEJ. Loss of SETD1A/BOD1L function induced uncontrolled DNA end resection, impaired end-joining of dysfunctional telomeres, and reduced immunoglobulin class switching, all of which are characteristic of 53BP1-RIF1 deficiency^[10]^. This is dependent upon lysine methylation by SETD1A, as these phenotypes were also apparent in cells deficient in SETD1A activity, in H3K4 methylation or overexpressing the H3K4 demethylase KDM5A. Furthermore, RIF1 and H3K4me3 overlap at a genome wide level, which seems independent of external factors including origin firing or transcription start sites. Therefore, H3K4 methylation seems to directly stimulate DSB repair by NHEJ. Interestingly, our data suggests that the mechanism by which H3K4me controls DSB repair is direct, as *in vitro* binding assays showed that RIF1 binds directly to methylated H3K4, an interaction mediated by the HEAT repeats present in the N-terminal of RIF1^[39]^. This is particularly intriguing given that similar experiments demonstrate that BRCA1 binds with a higher affinity to unmethylated H3 peptides compared to H3K4me3 peptides^[36]^, suggesting that H3K4me at DSBs might directly influence DSB pathway choice by regulating both BRCA1 and RIF1. Despite these advances, it is unclear exactly how H3K4me determines if a DSB undergoes repair by HR or NHEJ, and much work remains to identify the specific mechanism(s).

## TARGETING HR DEFICIENCY WITH POLY (ADP-RIBOSE) POLYMERASE INHIBITORS

Inherited mutations in either BRCA1 or BRCA2 are associated with susceptibility to multiple cancer types including a higher risk for breast and ovarian cancer. Since BRCA1 and BRCA2 regulate multiple stages of HR, cells with compromised BRCA1/BRCA2 activity are deficient in HR activity^[43]^. Targeting DSB repair deficiency represents an important paradigm in cancer therapy, exemplified by the use of poly (ADP-ribose) polymerase inhibitors (PARPi) to treat HR-deficient tumours^[44]^. PARPi work by trapping PARP enzymes on DNA, preventing the repair of single strand breaks (SSBs) via a PARP-reliant pathway known as base excision repair. As a consequence, unrepaired SSBs are converted into DSBs when encountered by replication forks. Since the resulting DSBs require repair via HR, in cells lacking sufficient levels of BRCA activity these DSBs cannot be repaired, resulting in NHEJ-dependent toxic chromosome fusions which drive cell death^[44,45]^. To date, four PARP inhibitors have received clinical approval in multiple BRCA1- and BRCA2-deficient settings: olaparib, rucaparib, talazoparib and niraparib.

Although treatment with PARPi induces a significant increase in patient survival, many patients develop resistance, and their prognosis is poor. Indeed, 40% of metastatic breast cancer patients harbouring germline BRCA1/2 mutations failed to respond to olaparib^[46]^. This resistance seems to arise from 4 main biological mechanisms^[47]^: restoration or reactivation of BRCA1 or BRCA2 activity (e.g., by reversion mutations or promoter demethylation); loss of PARP1 or PARG expression; upregulation of PARPi efflux; and rewiring of the DDR, including restoration of HR and replication fork protection. In particular, loss of members of the 53BP1-dependent NHEJ pathway (e.g., RIF1, REV7, 53BP1, Shieldin) renders BRCA1-deficient cells resistant to PARPi^[9,13,17,21]^. This is thought to be mediated via the absence of the Shieldin complex on DNA ends, leaving them unprotected and subject to resection by nucleases to initiate repair by HR^[48]^. Therefore, the balance between HR and NHEJ is key in determining the response to these targeted inhibitors. Interestingly, this mechanism of resistance has not been observed in BRCA2-deficient cells to date, which is likely due to differing roles between BRCA1 and BRCA2 in promoting HR^[49]^.

## SETD1A AND H3K4ME IN PARP INHIBITOR RESISTANCE

**Figure 1:**
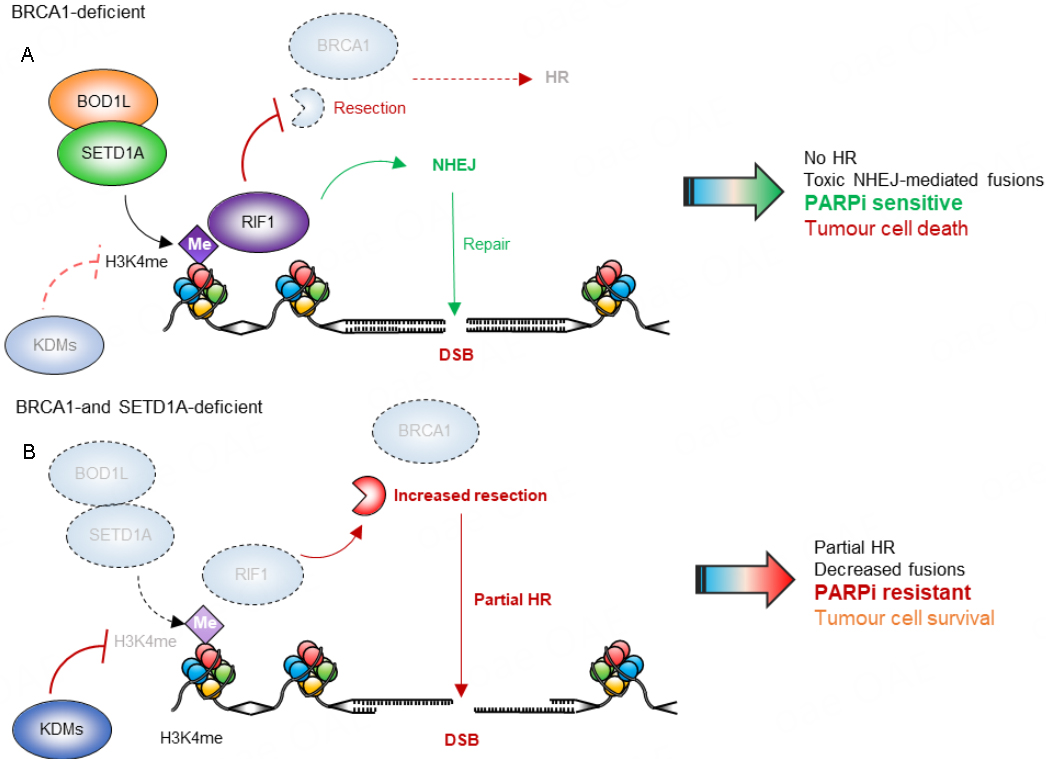
Effects of BOD1L/SETD1A loss on PARP inhibitor sensitivity in BRCA1-deficient cells. (A) H3K4me mediated by the BOD1L/SETD1A complex promotes RIF1 localisation at DNA double-strand breaks (DSBs) and stimulates NHEJ. In BRCA1-deficient cells, DNA-end resection and RAD51 loading are inhibited and lesions cannot be repaired by homologous recombination (HR), resulting in sensitivity to PARP inhibition and cell death. (B) Depletion of the BOD1L/SETD1A complex results in loss of H3K4me and decreased RIF1 localisation to DSBs. This allows DNA end-resection and RAD51 loading, partially restoring HR. This mediates resistance to PARP inhibition and allows cells to survive. Me: Methylation; PARP: poly(ADP) ribose polymerase; NHEJ: non-homologous end joining; KDMs: lysine demethylases.

Our recent findings impact substantially on these mechanisms of drug resistance. We demonstrated that, like loss of RIF1^[10]^, loss of SETD1A also induces PARPi resistance in BRCA1-deficient cells^[39]^. Our data also demonstrate that this resistance can be linked to a partial restoration of HR in these cells, as we observed cells deficient in both BRCA1 and SETD1A were able to recruit RAD51 to chromatin following treatment with PARPi, and that functional HR was at least partially restored in cells lacking both BRCA1 and SETD1A. Therefore, loss of SETD1A allows reactivation of HR in BRCA1-deficient cells [Figure 1]. Strikingly, many of these phenotypes (increased end-resection, defective RIF1 recruitment, PARPi resistance) were also observed in cells expressing SETD1A but in which H3K4 methylation had been perturbed by either mutation or over-expression of a lysine demethylase^[39]^, suggesting that PARPi resistance in BRCA1-defective cells is driven by epigenetic modifications, at least in part. Indeed, given that RIF1 interacts with H3K4me3 *in vitro*, this suggests that SETD1A-mediated histone methylation is responsible for promoting NHEJ and therefore sensitivity to PARPi [Figure 1]. 

Interestingly, SETD1A, H3K4me and RIF1 dysfunction is linked with a second mechanism known to control PARPi resistance, the protection of nascent DNA^[40,50]^. Newly replicated DNA is protected from degradation by several factors, including BRCA1, BRCA2, RIF1, 53BP1, SETD1A, BOD1L and H3K4me^[51]^, all of which also have roles in DSB repair. At stalled replication forks, these factors act to suppress the actions of nucleases including DNA2, EXO1 and MRE11. In their absence, excessive nucleolytic degradation leads to genomic instability and drives sensitivity to PARPi. Therefore, loss of fork degradation (or restoration of protection) leads to PARPi resistance^[52,53]^. However, this only seems to be applicable in certain genetic backgrounds: whilst loss of BRCA1/2 and thus loss of fork protection sensitises cells to PARPi, cells deficient of SETD1A, RIF1 or 53BP1 are also deficient in this pathway, but are not sensitive to PARPi. Furthermore, co-depletion of SETD1A and BRCA1, or RIF1 and BRCA1, does not restore fork protection^[39]^, suggesting a complex interplay between roles for these proteins at DSBs *vs.* replication forks. Nevertheless, our findings that PARPi sensitivity can be driven by epigenetic changes are in broad agreement with other studies demonstrating that such modifications can also regulate the response to PARPi^[53,54]^. Clearly, much more work remains to be done to comprehend the different mechanisms of DDR rewiring and how these impact on PARPi resistance in various genetic backgrounds and tumour types.

## THERAPEUTIC IMPLICATIONS AND FUTURE PERSPECTIVES

***Identifying resistance: *** In terms of clinical implication, predicting which patients may develop resistance to PARPi is an important area of investigation. Previous work has shown that significant changes in the expression and activity of methyltransferase and demethylase enzymes occurs during cancer development, suggesting that disruption to their function is important in disease pathogenesis^[55]^. Furthermore, analysis of publicly available datasets suggests that SETD1A expression correlates with chemotherapeutic sensitivity and overall survival in multiple tumour types^[56,57]^. This provides further evidence that SETD1A expression might be a useful prognostic indicator to be considered when choosing a patient’s treatment regime [Figure 1]. Furthermore, monitoring SETD1A expression during the onset of resistance, and linking BRCA1 mutation status with SETD1A expression, would be invaluable in evaluating its utility as a potential biomarker. Taken together, profiling of SETD1A expression may well be a valuable prognostic tool to identify patients who are more likely to develop resistance to PARPi allowing them to be placed on alternative therapies including KDM inhibitors or in combination other genotoxins, in the hope that this would kill resistant cancer cells.

**Figure 2:**
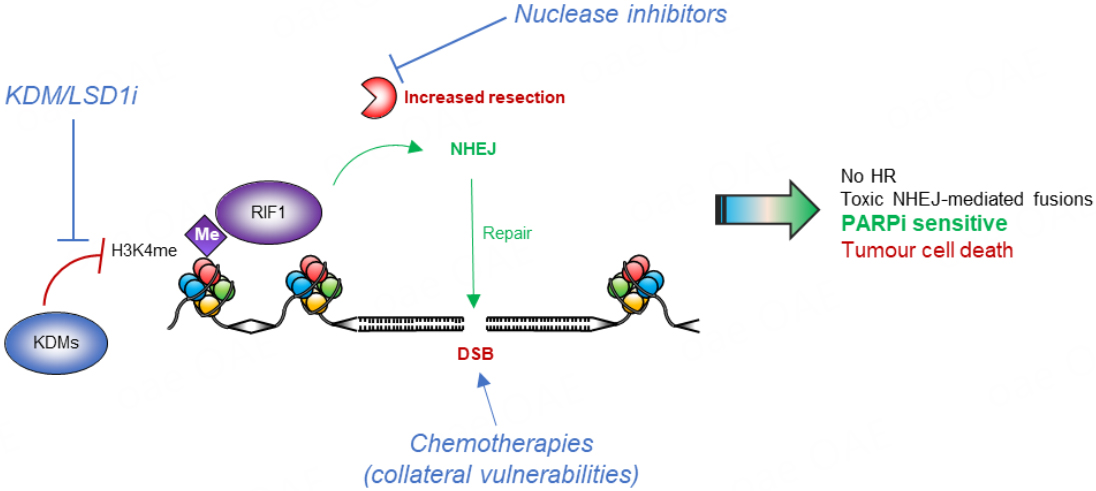
Future strategies to restore PARP inhibitor efficacy in BRCA1-deficient cells upon loss of SETD1A function. Sensitivity to PARP inhibition following loss of the BOD1L/SETD1A complex or H3K4me perturbation could potentially be restored via 3 mechanisms including: (1) inhibition of the lysine demethylases (KMD5 and LSD1) responsible for removing H3K4me; (2) inhibiting nucleases such as MRE11 to prevent DNA-end resection which facilitates HR; and (3) exploiting collateral vulnerabilities using chemotherapeutics, e.g., cisplatin. Me: Methylation; PARP: poly(ADP) ribose polymerase; HR: homologous recombination; NHEJ: non-homologous end joining; DSB: double-strand break; KDMs: lysine demethylases.

***Therapeutic approaches: *** Investigating novel ways of manipulating DSB repair is crucial for the development of new and more effective treatments for patients treated with PARPi [Figure 2]. Several potential strategies could be envisaged to prevent HR reactivation upon PARPi-resistance, ultimately increasing the efficacy of these therapies. Firstly, increasing H3K4me could represent a direct approach to facilitate RIF1 recruitment to DSBs, promoting NHEJ and driving toxic chromosomal fusions and cell death. This could be achieved via manipulating SETD1A expression/activity, or the inhibition of KDM enzymes to prevent the removal of specific methylation marks. KDM1A/LSD1 is a prominent demethylase which counteracts the activities of SETD1A, and inhibitors to this protein have already been developed^[58,59]^ and are currently being assessed for their use in cancer therapy. This raises the possibility that alleviating PARPi by manipulating the balance between H3K4 methylation and demethylation using these inhibitors could offer potential treatment benefit. Further pre-clinical work leading to their exploration in BRCA1-deficient patients would be an exciting avenue of future research.

A second approach to prevent reactivation of HR would be to inhibit the cellular nucleases responsible for DNA resection [Figure 2]. Loss of the 53BP1 pathway and/or SETD1A in BRCA-deficient cells allows uncontrolled end-resection by nucleases such as MRE11, CtIP, EXO1 and DNA2^[39]^. Combining PARPi treatment with inhibitors of these nucleases could be a promising way of preventing HR reactivation. Indeed, there is already evidence from pre-clinical studies that MRE11 inhibitors sensitise cancer cells to other agents such as IR^[60]^. Furthermore, MRE11 activity determines the sensitivity of cells to PARPi treatment in colorectal cancer^[61]^. However, given the diverse roles of MRE11 it would be important to monitor effects of its inhibition to ensure functions aside from its role in DNA end-resection are not compromised. CtIP depletion has also been shown to sensitise breast^[62]^ and ovarian^[63]^ cancer cells to treatment with PARPi, however this appears independent of BRCA-deficiency. As above, this opens novel areas of exploration, and could provide benefit to treat tumours without the traditional “BRCA-deficient” definitions.

Thirdly, deficiencies in pro-NHEJ components drive PARPi resistance in BRCA1-deficient cells, but also induces collateral vulnerabilities to other DNA-damaging agents including IR and cisplatin^[17]^. Exploring how loss of SETD1A/H3K4me affects the response to other genotoxic agents may help to identify other therapies that could be used to bypass PARPi resistance [Figure 2]. Notably, loss of SETD1A or its cofactor BOD1L sensitise cells to inter-strand crosslink (ICL)-inducing agents similar to cisplatin^[40]^. Furthermore, combinations of PARPi with pharmacological inhibitors to histone deacetylases, apical DNA repair kinases ATM and ATR, PI3K and mTOR, and immune checkpoint proteins have all been studied extensively^[64]^, and may provide worthwhile avenues of investigation in cells lacking SETD1A, BOD1L or H3K4me. Finally, exploring the link between SETD1A and H3K4me with MMEJ may offer an alternative therapeutic vulnerability^[65]^.

***Beyond BRCA: *** There is growing evidence that the efficacy of PARPi as an anti-cancer therapy extends beyond BRCA1/BRCA2-deficiency to a range of other factors involved in HR. For example, loss of other key HR pathway proteins such as RAD51 and PALB2, as well as the apical DNA repair kinase ATM, gives rise to synthetic lethality with PARP inhibition^[66]^. However, the mechanisms of resistance applicable to these contexts have not been widely explored. Previous studies have indicated that DDR rewiring is unable to restore HR in BRCA2-deficient cells. For example, depletion of 53BP1 cannot rescue HR in BRCA2-deficient mouse embryonic fibroblasts^[49]^. To date, known PARPi resistance mechanisms in BRCA2-deficient cells include loss of the PARG glycosylase^[67]^ and restoration of functional BRCA2 activity via the acquisition of secondary reversion mutations^[68]^. This suggests that the resistance mechanisms acting in BRCA2-deficient cells differ significantly to those in other HR-deficient contexts. A key area for future investigation is therefore to establish whether PARPi resistance induced by loss of SETD1A provides a general mechanism of resistance that can be applied to wider HR-deficient contexts including RAD51, ATM and possibly BRCA2 deficiency. Combined with the above developments, this will increase the efficacy of DDR inhibitors in the clinic and help develop novel biomarkers and treatment strategies to overcome resistance.

## CONCLUSION

The induction of DSBs by chemo- and radio-therapy has been used for many years in order to successfully treat a range of different cancers. However, the one major disadvantage of this approach is its lack of specificity. More recent developments involving the use of targeted inhibitors of DSB repair pathways such as PARPi have enabled more selective targeting of cancer cells, exploiting their intrinsic vulnerabilities such as HR deficiencies in BRCA-mutated cancers. However, these approaches are hampered by resistance. Our recent findings^[39]^ have added to this field by identifying the potential clinical usefulness of regulating RIF1-dependent NHEJ through manipulation of SETD1A-dependent H3K4me. This is of particular relevance in BRCA1-deficient patients who develop PARPi resistance in the clinic, as maintaining H3K4 methylation/SETD1A activity and therefore the recruitment of RIF1 to DSBs could be a key strategy to prevent treatment resistance in these patients. Despite these advances, there is still much work to be done in the fields of SETD1A, NHEJ and histone methylation to enable the development of more tailored treatments to eradicate human cancers.

## DECLARATIONS

### Copyright

© The Author(s) 2023.
